# Meningioma Mimicking Fibrous Dysplasia

**DOI:** 10.7759/cureus.5782

**Published:** 2019-09-27

**Authors:** Nur Afiah Kamaluddin, Ahmad Hadif Zaidin Samsudin, Wan-Hazabbah Wan Hitam, Mohtar Ibrahim

**Affiliations:** 1 Ophthalmology, School of Medical Sciences, Universiti Sains Malaysia, Kubang Kerian, MYS; 2 Radiology, School of Medical Sciences, Universiti Sains Malaysia, Kubang Kerian, MYS

**Keywords:** proptosis, meningioma, fibrous dysplasia

## Abstract

Despite being among the common primary intracranial tumors, intraosseous craniofacial meningioma is the least common subtype of meningioma accounting for only 1-2% of intracranial meningiomas. Interestingly, it can display clinical and radiologic features that can be confused for fibrous dysplasia. Scan imaging and biopsy are crucial for the diagnosis as well as for further proper treatment. We report a case of unilateral eye proptosis and optic neuropathy which was initially thought for fibrous dysplasia. Later the histopathology revealed meningioma grade 1. As the clinical presentations are almost undifferentiated, diagnosis and further prompt treatment are challenging.

## Introduction

Meningioma is a benign, neoplastic lesion which originates from arachnoid cells. It constitutes the most common tumor of the skull, with an incidence of 2.3/100,000 [1]. Scarcely, the tumor cells primarily spread within the cranial bone, followed by hyperostosis, making them classified as intraosseous meningiomas. It is predominant in middle-aged females [1]. Neurological symptoms such as headache and visual loss mostly depend on the location of the tumor. Hence, as a sphenoid bone meningioma is predominant, exophthalmos, visual impairment, and optic disc swelling are the most common presentations of the disease. A prompt and correct diagnosis is challenging in the predominant osseous lesion with minimal dural involvement. Thus, other relevant differential diagnoses such as fibrous dysplasia, Paget's disease of the bone of the skull, and benign osteosclerotic lesions such as osteoma are also in line. We describe a case of meningioma in an adult woman with the initial imaging highly suspicious of craniofacial fibrous dysplasia (CFD).

## Case presentation

A 48-year-old lady with underlying controlled hypertension presented to our eye clinic with the complaint of gradual protrusion of the left eye for the eight months which worsened in the past month. It is painless, but she experienced intermittent redness of the eye. Otherwise, there is no history of trauma and no reduced vision. Systemically it was unremarkable.

**Figure 1:**
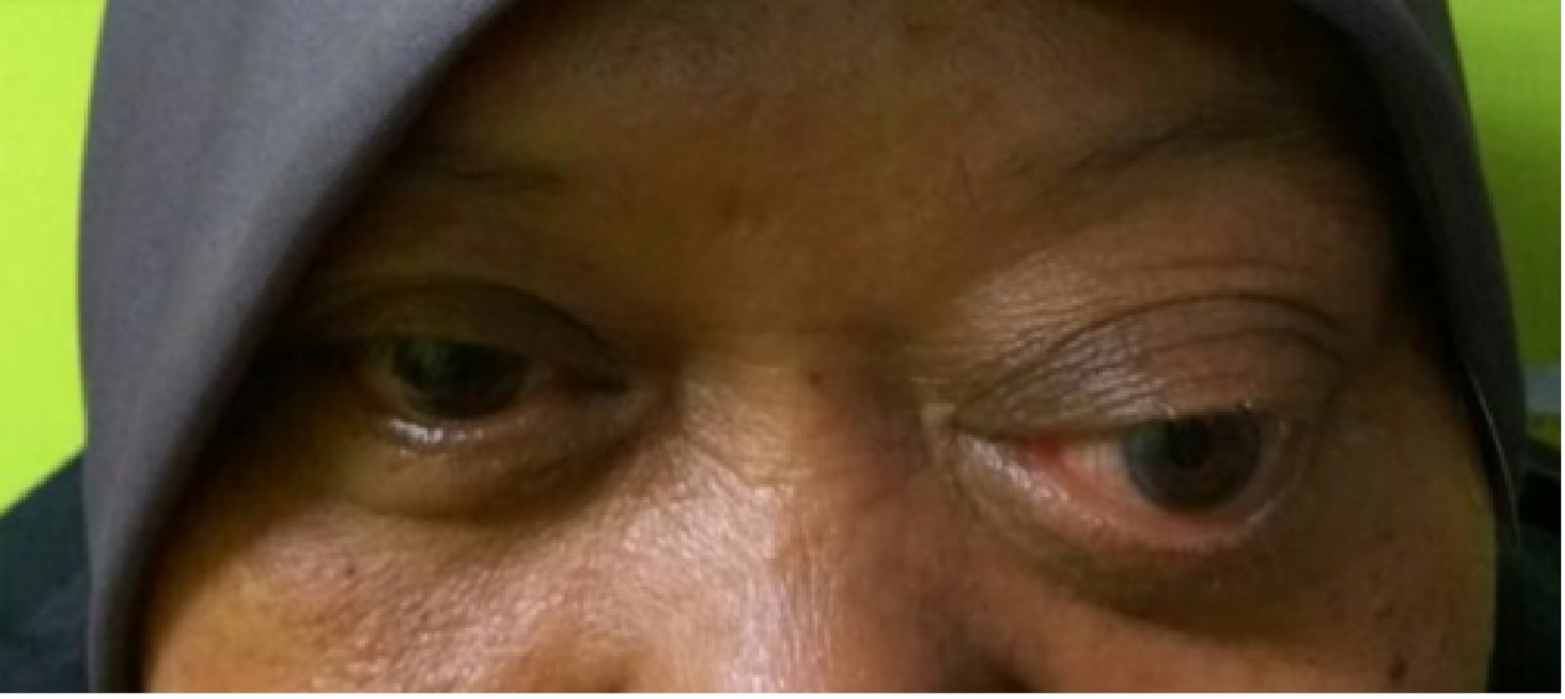
Marked proptosis over the left eye.

Visual acuity in the right eye was 6/7.5, and the left eye was 6/9. There was a presence of left relative afferent pupillary defect. Mild restriction in all directions of gaze was seen on the left side, as well as a relative proptosis of five millimeters as compared to the right eye (Figure 1).

**Figure 2:**
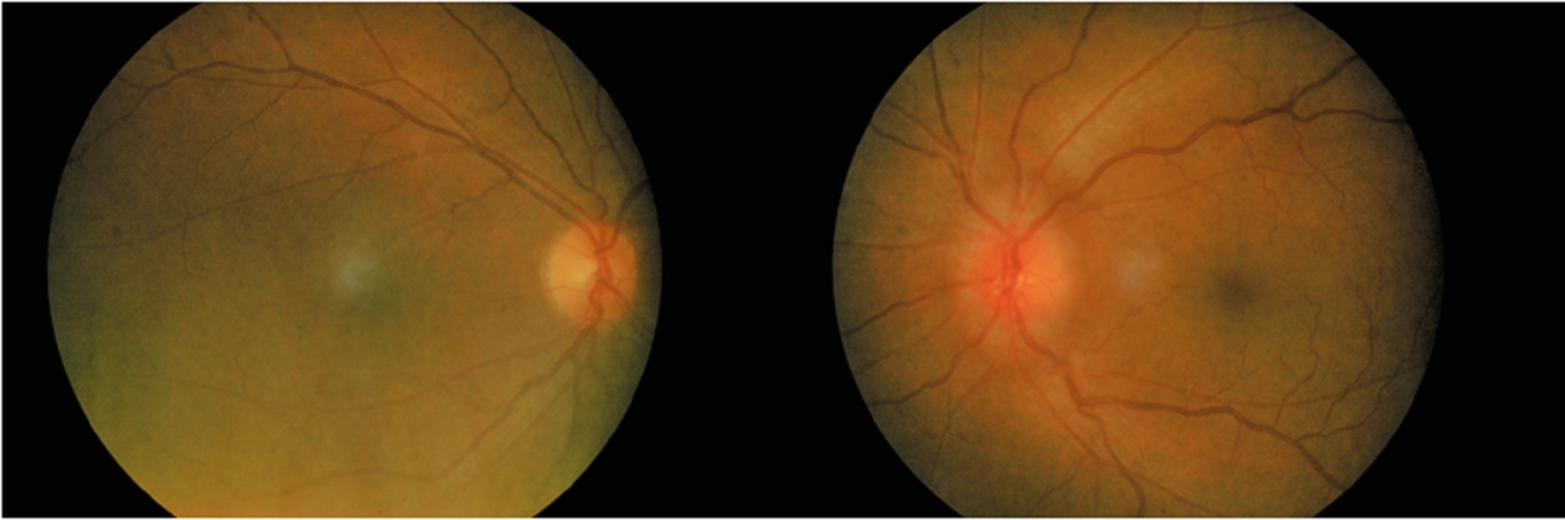
Fundus photo showing left optic disc swelling with mild tortuous and dilated vessels.

Optic nerve function tests and anterior segments were normal in both eyes. The intraocular pressure was normal. Fundoscopy of the left eye showed swelling of optic disc with tortuous and dilated vessels (Figure 2).

There was no peripapillary haemorrhages or choroidal folds. The macula was normal, and the retina was flat. The right eye fundus was normal. Humphrey visual field demonstrated a left eye inferior field defect. Other neurological examinations were normal.

**Figure 3:**
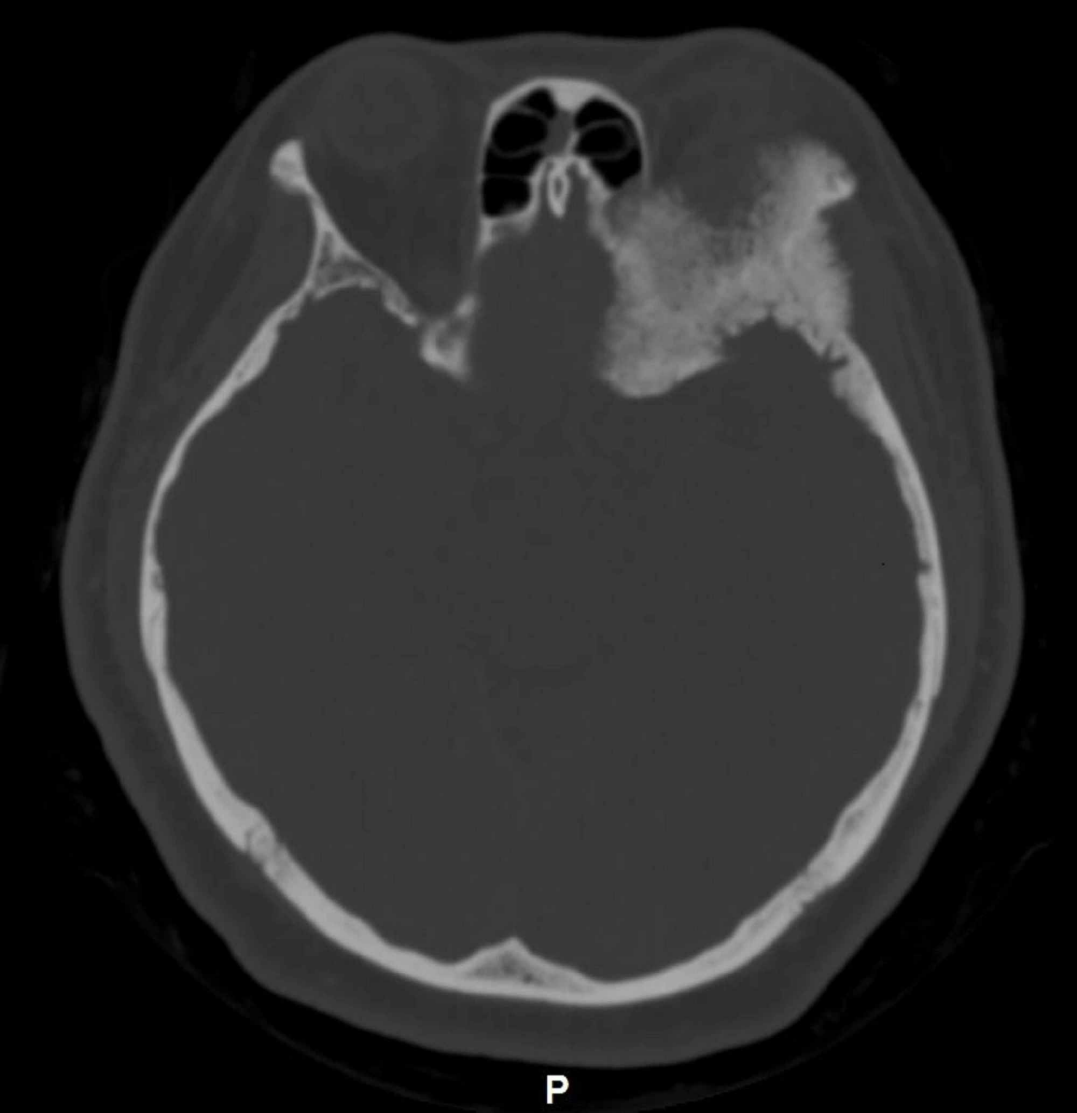
CT brain showing expansile osteoblastic lesion with irregular surface involving left greater wing of sphenoid and left zygomatic arch.

CT scan of the brain revealed the presence of an expansile osteoblastic bony lesion involving the left greater wing of sphenoid and left zygomatic arch (Figure 3).

**Figure 4:**
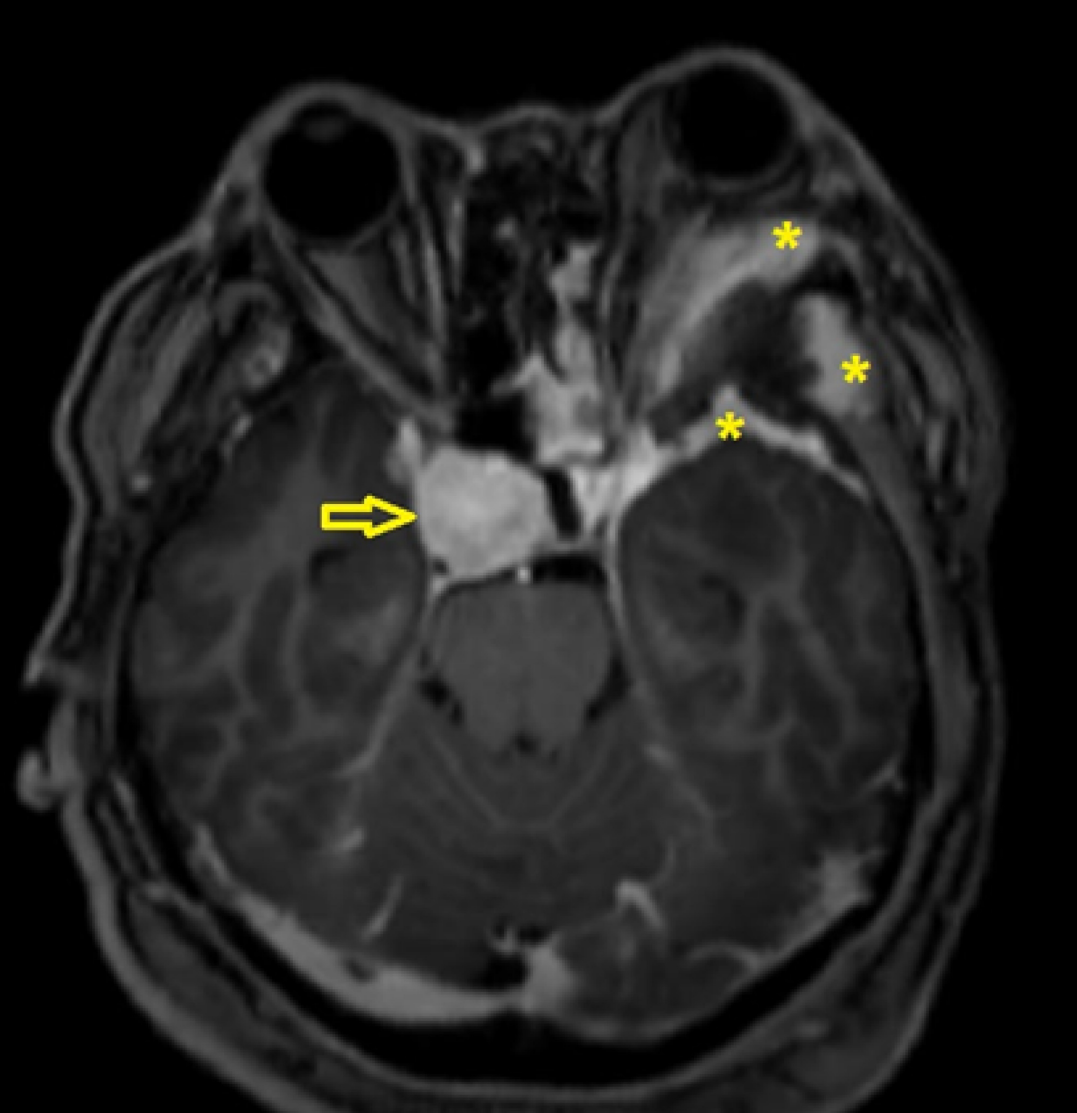
MRI with gadolinium showing enhancing soft tissue surrounding the lesion (*). Left orbital content is being pushed anteriorly. There is also presence of another lesion at parasellar region which showed vivid enhancement (open arrow). Both lesions are not in continuity with one another.

The bony lesion showed ground glass appearance with an area of irregular surface. It also associated with enhancing soft tissue mass. The lesion caused mass effect, pushing the orbit and its content anteriorly. At the same time, there was also another well-defined homogenously enhancing lobulated right sellar mass with extension into right parasellar region. On MRI, the lesion followed grey matter intensity on T1 and T2 weighted images and vividly enhanced post-contrast, suggesting meningioma (Figure 4).

Later she developed compressive optic neuropathy with worsening of vision, diplopia, and impaired optic nerve function test. She agreed for medical and surgical decompression. Intravenous methylprednisolone was given 1 g per day for three days before proceeding with the endoscopic orbital and optic nerve decompression and incisional biopsy. Another course of methylprednisolone was given 1 g per day for three days post-operatively. Subsequently, her symptoms improved well. HPE revealed benign meningioma with WHO grade 1.

## Discussion

Our patient presented with unilateral gradual exophthalmos, swollen optic disc, and later worsened with optic nerve decompression. Initial presentation is compatible with either CFD or meningioma; however, the initial scan is evocative of the CFD.

CFD is a benign condition where the fibrous tissue and woven bone replace the normal bone and marrow. The most common locations are the craniofacial bones, rib, and proximal femur, which the craniofacial region, particularly the anterior cranial base, is involved in over 90% of cases [2]. It usually occurs in the younger age group, although the disease may progress or manifest later in life. Primary bone tumors of the orbit represent a small percentage of all orbital tumors (0.6-2%), and the most commonly found were fibrous dysplasia and osteoma [3]. It usually prevails in children and early adolescents with some manifest in adulthood compared to predominantly adulthood in meningioma with a woman predilection in both diseases [4].

Contrarily, while meningiomas represent almost 15% of all brain tumors [5], intraosseous meningiomas are a rare subtype. It accounts for less than two percent of all meningiomas. It arises from meningocytes or arachnoid cap cells which entrapped in bone. They commonly demonstrate osteoblastic activity with hyperostosis surrounding a central lesion [5]. Most common symptoms include proptosis, visual loss, headache, ptosis, diplopia, and seizures. Painless progressive visual loss is due to the narrowing of the optic foramen [6].

Intraosseous meningioma (IM) may show osteoblastic, osteolytic, or mixed appearance on CT scan. While slightly less commonly encountered, osteolytic IM lesion is usually associated with intraosseous mass and is more likely to be malignant meningioma [7]. Commonly IM will present with osteoblastic appearance causing hyperostosis of the bone, which may be confused with fibrous dysplasia [7,8]. Both lesions can appear as ground-glass appearance. However, few features would suggest meningioma rather than fibrous dysplasia. These features include irregularity of the inner table, inward bulging to the intracranial, presence of soft tissue enhancement, adjacent cerebral edema, and subdural ossification [5,8,9]. Other differential diagnoses for osteoblastic lesion also include osteoma, metastasis, granuloma and Langerhans histiocytosis. The definite diagnosis obtained from the histopathological sample. In WHO criteria for meningioma grading, the grade 1 also known as benign meningioma will reveal histological variant most commonly meningothelial, fibrous, and transitional meningioma, and lacks criteria of atypical and anaplastic meningioma [10].

As the optic foramen is gradually narrowed, compression of the optic nerve is inevitable. Therefore, it is strongly recommended to decompress the optic canal in all involved cases to at least preventing more significant loss of vision [6]. In advance, radical resection of the tumor, the invaded bone as well as the dura mater is essential to correct the symptoms and lower the recurrence rate. However, the recurrence rate was still high, at 35-50%. This is due to the failure of early diagnosis, inadequate resection because of the involvement of the neurovascular structure, and also fear of the surgeons into iatrogenic death and severe complication in radical resection [6]. Furthermore, some require orbital reconstruction [6], and others will proceed with radiation therapy [11].

## Conclusions

Even with the typical presentation of fibrous dysplasia, a high level of suspicion for intraosseous meningioma is crucial, especially in regards to the sphenoid bone mass. This is to ensure early diagnosis, and further prompt treatment emerges. Biopsy and optic nerve decompression are vital nearly in all cases, and further radical resection is essential to prevent a recurrence.
